# Evaluating the Efficacy, Safety, and Patient Selection Criteria for Radiation Therapy in Early-Stage Dupuytren’s Contracture

**DOI:** 10.7759/cureus.112154

**Published:** 2026-07-06

**Authors:** Sukethram Sivakumar, Ryan Shah, Shray Jain, Nilanjan Haldar, Wenyin Shi

**Affiliations:** 1 Department of Radiation Oncology, Drexel University College of Medicine, Philadelphia, USA; 2 Department of Radiation Oncology, Thomas Jefferson University Hospital, Philadelphia, USA

**Keywords:** dupuytren’s contracture, non-surgical treatment, palmar fibromatosis, radiation therapy, radiology

## Abstract

Dupuytren’s contracture (DC) is a fibroproliferative disorder characterized by progressive contractures of the hand. The abnormal thickening of the palmar fascia can significantly impair hand function and lead to progressive pain, significantly reducing patients’ quality of life. Surgical interventions are commonly employed for moderate to severe cases and have long been considered the gold standard. Surgery, however, is often associated with morbidities, including complications, infection, and a significant rate of recurrence. Modern-day radiation therapy (RT) has emerged as a promising, though still largely investigational, option for selected patients with early-stage DC, with reported benefits in symptomatic control and disease stabilization. The objective of this review is to evaluate the literature supporting the safety and efficacy of RT techniques in the treatment of DC.

A literature review was conducted on March 13, 2025, using PubMed and Google Scholar with the search terms Dupuytren’s contracture, Dupuytren contracture, radiotherapy, radiation, radiation therapy, non-surgical, and therapy. Randomized controlled trials, retrospective cohort studies, and case series evaluating RT for DC were included, whereas reviews and studies lacking relevant clinical outcomes were excluded. Of 72 identified records, 29 full-text articles were assessed for eligibility, and 11 studies were included in the final review. Study selection and full-text review were performed by a single reviewer, and reported rates of disease control, progression, and treatment-related toxicities were compared across studies.

Overall, the literature suggests that RT may provide meaningful symptom control and disease stabilization with a generally favorable toxicity profile. Chronic toxicities appear uncommon and are usually limited to mild skin changes. RT appears generally well tolerated and potentially useful in selected patients with early-stage DC, although definitive long-term safety and its impact on reducing subsequent invasive interventions remain uncertain.

## Introduction and background

Dupuytren’s contracture (DC) is a fibroproliferative disorder characterized by progressive hand contractures, mostly affecting the palmar fascia of the fourth and fifth fingers. DC affects approximately 5% of the global population, with a higher prevalence in males. Common risk factors for DC include advanced age, North European ancestry, family history, smoking or tobacco use, and diabetes [1]. The precise etiology of the condition remains unclear, though the prevailing theory suggests that dysregulation of the Wnt pathway contributes to myofibroblast activation and fibrosis in genetically predisposed individuals [2].

The abnormal thickening of the palmar fascia leads to significant impairment in hand function, often necessitating surgical intervention in severe cases. Diagnosis can be facilitated using the tabletop test, in which a patient is asked to place their hand flat on a table with the palm facing downward; an inability to do so is indicative of DC. The condition is characterized by the formation of nodules beneath the skin, accompanied by cords, which are thickened bands of tissue that may cause the fingers to contract. These structures are primarily composed of type III collagen, leading to the development of dense, rope-like formations beneath the skin of the palm. DC is primarily diagnosed clinically through physical palpation of characteristic palmar nodules or cords and by a positive tabletop test.

DC progresses through three distinct stages: proliferative, involutional, and residual [3]. During the proliferative stage, myofibroblasts and fibroblasts are arranged in a spiraled pattern, contributing to excessive extracellular matrix production. In the involutional stage, fibroblasts align along the longitudinal axis of the hand, promoting tissue contraction. Finally, in the residual stage, the collagen-rich cords become fully developed, resulting in varying degrees of contracture and functional impairment. DC is classified into stages ranging from Stage 0 to Stage 4. Stage 0 indicates no palpable lesions, while Stage N signifies a palmar nodule without contracture. Stages 1 through 4 denote increasing degrees of flexion deformity: Stage 1 (0° to 45°), Stage 2 (45° to 90°), Stage 3 (90° to 135°), and Stage 4 (greater than 135°).

Treatment for DC varies depending on disease severity, with mild cases often requiring no intervention. However, moderate to severe cases typically require surgical intervention, such as limited fasciectomy or percutaneous needle fasciotomy (PNF), or injections with corticosteroids or collagenase clostridium histolyticum (CCH). Limited fasciectomy is the gold standard treatment for moderate to severe contractures in surgical candidates desiring a durable outcome. Limited fasciectomy has a relatively low recurrence rate of 3-15% at 2-3 years but carries higher risks compared with less invasive options, including a 5% rate of serious complications such as nerve injury, infection, and complex regional pain syndrome [4].

PNF offers a less invasive surgical intervention for patients with mild to moderate contractures. However, reported PNF recurrence rates range from 30% to 50%, though major complications are rare (<1%) [4,5]. CCH injections are suitable for patients with a palpable cord and a preference for nonsurgical treatment. Typically used for less severe DC, CCH has a recurrence rate of 15% to 55% and is generally associated with a low rate of serious complications; common adverse events include skin tears and swelling, while tendon rupture is rare. Steroid injections are used primarily in early nodular disease without fixed contracture, especially in the presence of pain or rapid disease progression. They provide less durable control and have less utility in advanced DC, and steroid injections are associated with approximately 50% recurrence or reactivation of nodules within 1-3 years [4]. The most common side effects are local skin atrophy and depigmentation. Given the challenges of DC recurrence and the higher risks associated with surgical intervention, there is a critical need for alternative, noninvasive treatment options for DC.

Concerns regarding potential late radiation effects and radiation toxicity have historically limited the widespread adoption of RT for this benign condition. However, in recent years, radiation therapy (RT), particularly electron beam RT, has become a promising noninvasive alternative for the management of early-stage DC due to its ability to reduce fibroblast and myofibroblast proliferation while modulating inflammatory pathways. Notably, the relatively low risks of RT and low recurrence rates have made this treatment an appealing method for managing early-stage DC and preventing the need for more invasive interventions. RT slows disease progression by reducing local inflammation [6]. Radiation-induced free radicals further suppress the proliferative activity of these cells, slowing disease progression. Controlled, low-dose RT has been shown to alleviate symptoms and, in some cases, stabilize or reduce early nodular disease. Despite this, the role of RT in DC remains controversial because of limited randomized evidence, uncertainty regarding long-term safety, and ongoing debate regarding the use of RT in treatment. The objective of this review is to evaluate the literature evidence supporting the safety and efficacy of RT techniques in the treatment of DC.

## Review

Methods

**Figure 1:**
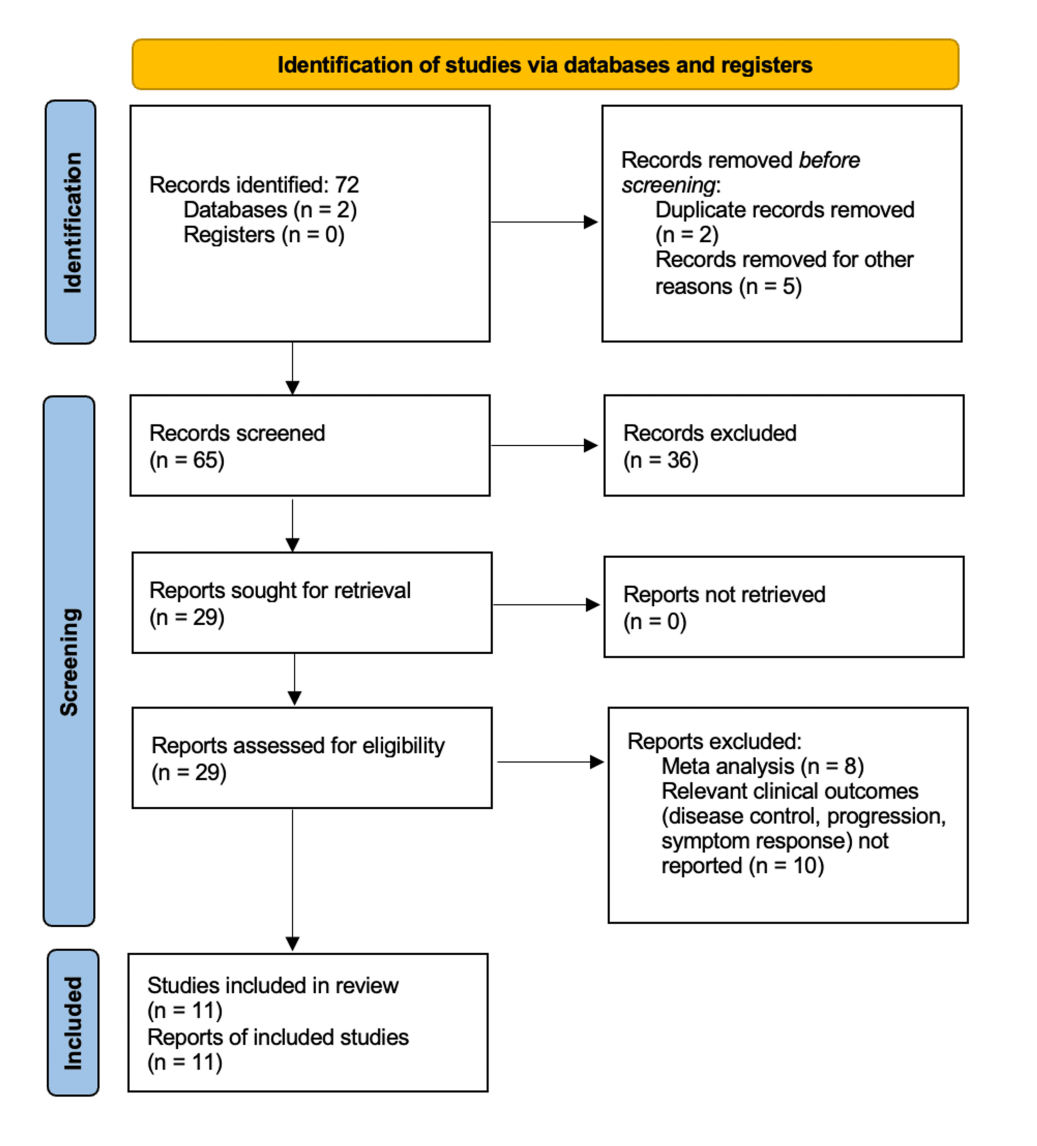
Identification of studies for radiation therapy in Dupuytren’s contracture.

An electronic literature search was conducted on March 13, 2025, using PubMed and Google Scholar to identify studies meeting the predefined eligibility criteria. The search strategy incorporated combinations of relevant keywords, including ("Dupuytren's contracture" OR "Dupuytren contracture") AND ("radiotherapy" OR "radiation" OR "radiation therapy") AND ("non-surgical" OR "therapy"). For Google Scholar, the first 100-200 results sorted by relevance were screened for eligibility. Searches were limited to English-language studies involving human subjects. These terms were applied to titles and abstracts to capture relevant studies. Of 72 identified records, 29 full-text articles were assessed for eligibility, and 11 studies were included in the final review (Figure 1). No automated tools were used for study selection. Duplicate records were removed manually prior to screening. This review was not conducted as a formal Preferred Reporting Items for Systematic Reviews and Meta-Analyses (PRISMA) systematic review, but rather as a structured narrative literature review.

Inclusion criteria were defined as follows: (1) adult patients diagnosed with DC; (2) randomized controlled trials, retrospective cohort studies, and case studies with no specific date range; (3) radiotherapy limited to electron, orthovoltage, and soft X-ray RT; and (4) studies reporting fractionation methods, disease control, or disease progression.

Exclusion criteria included: (1) meta-analyses and systematic reviews; (2) studies using non-standard radiation techniques, such as brachytherapy or proton therapy; (3) unpublished studies; (4) studies in non-English languages without English translation; and (5) studies lacking relevant clinical outcome data.

The initial literature search was performed by a single author, with eligibility determined through title and abstract screening. Full-text reviews were subsequently performed by the same author. As study selection and full-text review were conducted by a single reviewer, there is a potential risk of selection bias. Data extracted from the included studies included study design, patient characteristics, treatment parameters, and reported clinical outcomes. No formal risk-of-bias assessment was performed. Where reported, adverse event data were extracted as described by the original authors; “not available” indicates absence of reporting in the study, while “not reported” indicates that the outcome was not assessed. Due to heterogeneity across study designs, populations, follow-up durations, and outcome measures, including disease stability, symptom improvement, progression, and recurrence, results were synthesized descriptively without quantitative pooling. Conclusions were drawn through qualitative synthesis of reported trends in disease stability, progression, and toxicity across studies.

Clinical evidence

The efficacy of RT in the treatment of DC has been evaluated through randomized clinical trials, retrospective cohort studies, and case series. This review of RT for DC identified 11 studies spanning from 1985 to 2025, most of which utilized a regimen of 3 Gy per fraction over either 7 or 10 fractions (Table 1). Regimens of 10 × 3 Gy (30 Gy) were delivered over two 15 Gy courses with a 6-8-week break interval, a split-course approach designed to allow normal tissue recovery and minimize toxicity while maintaining therapeutic effects. In contrast, regimens of 7 × 3 Gy (21 Gy) required only one course of treatment. The study by Meredith R et al. primarily evaluated radiation dosimetry and treatment planning rather than clinical efficacy outcomes.

**Table 1:** Summary of literature evaluating radiation therapy for Dupuytren’s contracture. DC: Dupuytren’s contracture; RT: Radiation therapy.

Study	Year	Patients	Technique	Dose per fraction (Gy)	RT fractionation	Efficacy	Adverse events	Key limitations
Randomized controlled trials								
Seegenschmiedt MH et al. [7]	2001	N = 129	Orthovoltage RT	3 Gy	7 or 10 fractions	Overall reduction in symptoms, nodules, and cords; less than 10% of patients exhibited progression	Acute toxicity was minimal, mainly skin reactions and dryness; skin atrophy was uncommon	Single-center study; limited follow-up beyond 12 months
Meredith R et al. [8]	2017	N = 10	Electron beam RT (6 MeV)	3 Gy	10 fractions	Dosimetric comparison only; clinical efficacy outcomes were not reported	Not applicable	No clinical outcome data; purely dosimetric study; small sample
Retrospective cohort studies								
Herbst M and Regler G [9]	1985	N = 33	Orthovoltage RT	3 Gy	10 fractions	98% of patients reported no progression	None reported	Oldest study; no disease staging; no long-term follow-up reported
Keilholz L et al. [10]	1996	N = 96	Orthovoltage RT	3 Gy	10 fractions	At 3-month follow-up, 92% remained stable, 7% improved, and 1% progressed; 23% of patients reported progression at 5-year follow-up	Not available	Heterogeneous follow-up; no standardized outcome instrument
Adamietz B et al. [11]	2001	N = 99	Orthovoltage RT	3 Gy	10 fractions	84% of patients remained stable when RT was applied in earlier phases	Not available	No long-term follow-up
Betz N et al. [12]	2010	N = 135	Orthovoltage RT	3 Gy	10 fractions	Stable response in 59% of patients; improvement in 10% of patients	Not available	No staging information
Zirbs M et al. [13]	2015	N = 206	Soft X-ray RT	4 Gy	8 fractions, split course	45% of patients reported regression of symptoms	None reported/not available	Non-standard technique; no long-term follow-up
Schuster J et al. [14]	2015	N = 33	Electron beam RT (6-12 MeV)	3 Gy	7 or 10 fractions	Patient-reported overall treatment success in 31 of 33 patients	No grade ≥2 late toxicities reported	Very small sample; follow-up not reported; no formal outcome instrument
Smith ML et al. [15]	2015	N = 31	Electron beam RT (6-12 MeV)	3 Gy	7 fractions	100% of patients reported reduction or complete disappearance of pain after RT	50% reported acute toxicity, with the majority having mild symptoms, including skin tenderness and redness	Single institution; patient-reported pain outcomes only; no long-term follow-up
Case series								
Solie P et al. [16]	2023	N = 2	Electron beam RT (6-9 MeV)	3 Gy	10 fractions	Resolution in both patients	Not available	No generalizability; no formal follow-up; adverse event data not reported
Adjuvant RT								
Burgess T et al. [17]	2025	N = 60	Electron beam RT (6 MeV)	3 Gy	10 fractions	Not reported	44.1% of mild adverse events attributed to RT	Ongoing trial; efficacy data pending; small adjuvant arm; limited power to detect wound-healing complications

RT regimens involving 3 Gy per fraction, administered over 7 or 10 fractions, demonstrated significant efficacy, with rates of symptom stabilization, defined as the absence of clinical progression of nodules, cords, or contracture, ranging from 84% to 98%. However, outcome definitions were not uniform across studies and included measures of disease stability, regression, progression, and patient-reported symptom improvement. The best evidence comes from an RCT by Seegenschmiedt MH et al., which compared two RT dose-fractionation regimens rather than RT versus observation and therefore provides evidence regarding regimen selection. The study involved 129 patients with early-stage DC characterized from Stage N to Stage II [7]. Patients were randomized to either 30 Gy in 10 fractions, delivered as two 5 × 3 Gy series separated by 8 weeks (Group A), or 21 Gy in 7 fractions over 2 weeks (Group B). At 12 months, disease regression was observed in 56% of hands in Group A and 53% in Group B, disease stability in 37% and 38%, and progression in only 7% and 9%, respectively. At the one-year follow-up, no difference in response rates was observed between the groups. Most of the studies included in this review were retrospective cohort studies; similarly, these studies found that the majority of patients with early-stage DC reported clinical improvement, with only 14% to 31% experiencing disease progression [12].

Orthovoltage and electron beam RT were the predominant modalities, with earlier studies favoring orthovoltage and more recent studies opting for electron beam therapy. These findings were further supported by cohort studies by Schuster J et al. and Smith ML et al. [14,15]. While orthovoltage RT was historically favored, electron RT has become more widely adopted due to its ability to minimize the out-of-field dose and limit radiation exposure to deeper tissues. Although less commonly used, X-ray RT also yielded promising results, albeit with a distinct dose schedule. Contemporary superficial RT techniques may further improve tissue sparing through limited penetration to deeper hand structures. However, clinical experience with these approaches in DC has remained limited, and additional studies are needed to better characterize long-term outcomes.

Reported adverse events were generally minimal and included dryness, tenderness, and erythema. Long-term effects such as skin atrophy or excess fibrosis were rare among the cohorts and were explicitly reported as absent in most studies. An exception was noted in the study by Smith ML et al., where 50% of patients experienced acute toxicity, primarily mild skin symptoms. No instances of secondary malignancy following RT treatment of DC have been reported in the literature. However, this should be interpreted cautiously, as limited sample sizes, follow-up durations, and potential under-reporting may hinder the detection of rare late events. Collectively, these findings suggest that RT may provide clinical benefit in patients with early-stage DC, with generally low reported toxicity, although these conclusions should be interpreted in light of the predominantly retrospective design, heterogeneous outcomes, and limited long-term toxicity data.

Adjuvant RT

This section summarizes early safety data from an ongoing trial evaluating adjuvant radiotherapy; efficacy outcomes have not yet been reported. Adjuvant radiotherapy is currently being investigated as a postoperative or post-procedural intervention, but clinical efficacy has not yet been established. Limited data are available in the literature to support this discussion. The Dupuytren’s disease Evaluation of Preventative or Adjuvant Radiation Therapy (DEPART) trial is an ongoing randomized controlled trial that includes an adjuvant arm, with patients beginning adjuvant RT (30 Gy in 10 fractions) after limited fasciectomy, collagenase injection, or percutaneous needle fasciotomy [17]. Although efficacy data have not been fully reported, adverse event data for the adjuvant arm have been published. Over four years of follow-up, 114 adverse events were reported in the adjuvant RT group (n = 28). Of these, 102 (89%) were mild, and 45 (44.1%) of the mild events were attributed to radiotherapy, mostly dermatitis and reduced sweating. Twelve adverse events overall were moderate to severe, with 3 attributed to radiotherapy. The only persistent adverse event attributed to radiotherapy between 12 and 36 months was reduced sweating. No differences in wound complications were observed between the adjuvant RT group and surgical intervention-only groups in the available data, although the small sample size may limit the detection of uncommon wound-healing complications. As efficacy outcomes remain pending, no conclusions regarding the clinical effectiveness of adjuvant radiotherapy can be determined.

Patient selection

To date, no established guidelines exist for patient selection for RT in DC. However, the first known randomized clinical trial on this subject utilized the following inclusion criteria: age ≥36 years, clinically evident DC, and the ability to adhere to treatment protocols (Table 2). Patients with contraindications to RT, such as pregnancy, were excluded from the study. However, in the absence of standardized patient-selection criteria, the optimal candidates for RT remain uncertain, underscoring the need for further research to identify patients most likely to benefit from treatment.

**Table 2:** Patient selection and radiation therapy planning parameters in Dupuytren’s contracture. Source: Reference [7]. RT: Radiation therapy.

Patient selection	Patient setup	Dose	Assessment	Retreatment
- Age ≥36 years - Palpable cords/nodules without significant contracture - Preserved finger extension and range of motion - No contraindications to RT	- Treatment field restricted to the affected area(s) of the hand(s) - 1 cm medial and lateral margins - 1.5 cm proximal and distal margins - Minimum field size of 4 cm	- 30 Gy (3 Gy × 10 fractions, with an 8-12-week break after 15 Gy) - 21 Gy (3 Gy × 7 fractions), for patients preferring to avoid a split course	- Clinical evaluation after treatment - Reassessment at scheduled intervals to monitor progression	Based on initial response and determined on a case-by-case basis

Treatment recommendations

The assessment of DC typically begins with a referral from a hand surgeon or dermatologist. While the clinical presentation of DC is often distinctive, a thorough physical examination is essential to evaluate the extent of nodule formation, cord thickening, and functional impairment of the hand.

RT is primarily recommended in the early stages of DC, when fibroblast and myofibroblast activity can be modulated before irreversible contractures develop. Early intervention has been associated with higher rates of disease stabilization and reduced progression [18]. Early-stage DC is typically defined using the Tubiana scale as Stage N or N/I, characterized by palpable nodules or cords with minimal flexion deformity, approximately 1°-5° [10]. The treatment field is restricted to the affected hand to limit radiation exposure to surrounding tissues [19]. Technical treatment parameters, including dose, field design, fractionation schedules, and setup considerations, are summarized in Table 2.

Prior to initiating RT, patients should engage in a comprehensive discussion with their physician regarding the benefits and potential risks. The use of a bolus is considered on an individual basis, depending on clinical factors, with custom in-house 3D boluses being an option. The most common treatment regimens involve 3 Gy per fraction over 7 or 10 fractions. The 30 Gy split course shows slightly lower progression rates at five years (~19.5%) compared with the 21 Gy continuous course (~24%) but requires a longer treatment duration [19]. The more conservative fractionation approach also minimizes toxicity, though the difference between regimens is slight. The 2015 DEGRO guidelines similarly recommend consideration of RT in selected patients with early-stage DC and provide recommendations regarding treatment fields and fractionation schedules [20].

Post-treatment, patients should be advised to apply moisturizer two to three times daily to minimize tenderness and skin peeling. Additionally, patients are recommended to use protective measures for the treated hand and avoid direct trauma to the area. While adverse effects can occur, they are generally mild, with dryness being the most common [21]. This risk is further mitigated by precise patient setup, including limiting radiation fields to the fourth and fifth digits. Given the potential for disease recurrence, patients should be advised to maintain regular follow-up and report any signs suggestive of DC.

Limitations

The available evidence evaluating RT for DC is limited by the predominance of retrospective studies, heterogeneous outcome definitions, and variability in radiation protocols across studies. Most studies focused on early-stage disease, limiting generalizability to more advanced contractures. Additionally, this review used a single-reviewer screening process and did not perform a formal risk-of-bias assessment, introducing the potential for selection bias. Many included studies also had small sample sizes, limiting the statistical power and generalizability of reported outcomes. Furthermore, the literature search was limited to PubMed and Google Scholar, excluding other biomedical databases such as Embase or the Cochrane Library, which may weaken the comprehensiveness of the search strategy.

Variability in study design, patient populations, and outcome reporting should be considered when interpreting the findings of this review. Further prospective studies with standardized outcome measures and longer follow-up are needed to better characterize the long-term efficacy and safety of RT for DC.

## Conclusions

DC can lead to significant functional impairment, adversely affecting quality of life for many patients. While surgical intervention can provide durable control for moderate to severe cases, it is associated with prolonged recovery and significant risks compared with less invasive interventions. Injections with CCH or steroids can be useful in early-stage DC, but rates of progression and recurrence remain high. RT appears generally well tolerated and potentially useful in selected patients with early-stage DC, but definitive long-term safety and its impact on reducing later invasive interventions have not been established. Future studies should focus on standardizing treatment protocols and follow-up evaluation criteria to better characterize outcomes and optimize patient selection.
